# Engaging With a Community of Practice in Dementia: Impacts on Skills, Knowledge, Networks and Accessing Support

**DOI:** 10.1111/hex.70154

**Published:** 2025-01-15

**Authors:** Clarissa Giebel, James Watson, Megan Polden, Megan Readman, Hilary Tetlow, Mark Gabbay

**Affiliations:** ^1^ Department of Primary Care & Mental Health University of Liverpool Liverpool UK; ^2^ NIHR Applied Research Collaboration North West Coast Liverpool UK; ^3^ Division of Health Research Lancaster University Lancaster UK; ^4^ Department of Psychology Lancaster University Lancaster UK; ^5^ SURF Liverpool UK

**Keywords:** community of practice, dementia, public engagement

## Abstract

**Background:**

To deliver implementable, meaningful research and advance knowledge, different stakeholders need to be brought together regularly via a suitable platform or community of practice. The Liverpool Dementia & Ageing Research Forum, set up in 2019, is a public/professional community of practice, providing in‐person and remote events and activities to connect people living with dementia, unpaid carers, health and social care professionals, Third Sector representatives and commissioners. The aim of this study was to qualitatively explore the experiences and impacts of engaging with Forum events by different stakeholders.

**Methods:**

Attendees of any Forum events were eligible to take part. We conducted remote semi‐structured interviews about the experiences and impacts of engaging with the Forum between November 2023 and April 2024. Anonymised transcripts were coded by three research team members, including a trained public advisor (unpaid carer), using inductive thematic analysis.

**Results:**

Seventeen attendees participated in the study. These included people living with dementia, unpaid carers, Third Sector representatives, academics and health and social care providers. Four overarching themes were identified in the data: facilitated networking within and outside of personal expertise, improved knowledge and capacity, empowerment by having one's voice heard and diversity in stakeholders but not the background. The Forum events were utilised on a drop‐ in and ‐out basis for most attendees, often picking events that were of immediate interest to them, whilst some attended almost all events. Engaging with the Forum was found to strongly facilitate increased networking and building relationships, especially outside of stakeholder background, whilst diversity in backgrounds of attendees, such as ethnic diversity, was lacking.

**Conclusions:**

This Community of Practice has successfully brought together diverse stakeholders to network and expand their relationships, and increased knowledge and capacity in different stakeholder groups. Whilst the Forum was considered inclusive, future outreach work needs to ensure that greater diversity in backgrounds and personal experiences is reflected in attendees, and that a continuous flow of new attendees joins.

**Patient and Public Involvement:**

One unpaid carer was involved as a public advisor and was supported in coding two anonymised transcripts and interpreting the findings. They also helped codesign the semi‐structured topic guide.

## 1 Introduction

Dementia directly affects ~1 million people in the United Kingdom, with numbers estimated to rise to 1.4 million by 2040 [1]. Despite the growing number of people living with or caring for someone with the condition, there remains a notable lack of knowledge and understanding about dementia, different subtypes, and how to receive the correct diagnosis and suitable care [2, 3, 4]. This is the case for both people with lived experiences of dementia and the health and social care workforce, creating unnecessary barriers to receiving a timely and correct diagnosis and adequate care and support for many [5, 6].

One way to share and advance knowledge in any field is through communities of practice. Communities of practice, first termed by Lave and Wenger [7], bring together individuals within a profession, such as nursing or general practice, or around a topic area, such as dementia, learning disabilities or cancer, with the common goal of exchanging knowledge and developing a learning partnership. Besides advancing knowledge, social relationships can be built over time [8, 9, 10]. Whilst communities of practice may end up progressing into working together, they are not task‐focused but instead focused solely on the exchange of knowledge and experiences around a specific topic.

A dementia‐specific community of practice, the Liverpool Dementia & Ageing Research Forum, was set up in 2019 by a dementia researcher to bring together diverse stakeholders in dementia and ageing, including people living with and caring for someone with dementia, former unpaid carers, health and social care professionals, Third‐sector representatives, commissioners, academics and the general public. Third‐sector organisations are Charities and non‐profit organisations that provide information, care and/or support to people with dementia, unpaid carers and older adults. In the United Kingdom, these include Alzheimer's Society, Dementia UK and the Lewy Body Society. Commissioners include staff working in government organisations and smaller local government organisations, such as local authorities that commission and allocate social care. Anyone with an interest in the field can join the mailing list or sign up to attend public events. By bringing together diverse stakeholders via different regular in‐person and remote meetings and events, including regional (North West) networking meetings, bimonthly webinars, monthly journal clubs, an annual conference and other ad hoc events, such as hosting a Member of Parliament, the Forum offers the opportunity for different stakeholders to connect and to jointly co‐produce research. Early survey evaluation of the Forum has shown that it improves collaboration and networking among attendees, improves knowledge about dementia and available services, and has also led to access to care and support services for some people living with dementia [11]. Besides resulting in research grant applications, some of which have been funded, it has also led to the co‐production of a game about dementia inequalities, the Dementia Inequalities Game [12]. With the Forum active for several years, it is important to capture the nuanced experiences of attendees and their perceived impacts of engaging with the Forum on their skills, knowledge, and personal and professional topic‐specific networks. The Forum is hosted and supported by the National Institute for Health and Care Research Applied Research Collaboration North West Coast.

To date, only one other UK and one international community of practice in dementia have been described in the published research literature. Mayrhofer et al. [13] report on a community of practice for dementia champions across one county in the South East of England. Dementia Champions were professionals in different National Health Service Trusts across the county. The authors reported on a temporarily funded community of practice, which was only available for this single group of stakeholders and in one county. To ensure sustainability, they argue that different stakeholders need to be involved also. As no evaluation has taken place, the impacts of knowledge exchange, capacity building and social relationship building remain elusive for this reported community of practice in dementia. Moreover, Ong et al. [14] report on an international community of practice on environmental design in dementia, and reflections from very few members who are also co‐authors. These include how as a result of this special interest group, they were asked to contribute to design discussions around new signage in a public space. Considering that the Liverpool Dementia and Ageing Research Forum is bringing together diverse stakeholders regularly, primarily in the United Kingdom, and the lack of previous in‐depth qualitative evaluations of such a community on dementia, it is important to assess the Forum's impact.

Thus, the aim of this qualitative study was to evaluate the experiences and impacts of engaging with the Liverpool Dementia & Ageing Research Forum by different stakeholders. This model of outreach and public involvement and engagement may prove impactful on individuals and in connecting stakeholders with diverse personal, caring, professional and voluntary sector experiences of dementia. Findings from this dementia‐specific community of practice may be adaptable to other population groups and conditions.

## 2 Methods

### 2.1 Participants and Recruitment

People living with dementia, unpaid carers, health and social care professionals, academics, students, Third Sector representatives and commissioners living in the United Kingdom—all stakeholder groups engaging with the Liverpool Dementia and Ageing Research Forum, were eligible to participate if they had joined at least one Forum activity since its inception in 2019 and were aged 18+. There are no age restrictions for people wishing to attend the Forum, although no person under the age of 18 has to date attended any Forum events. Whilst the Forum is open to people across the world, due to ethical restrictions, we only included stakeholders residing in the United Kingdom. Ethical approval was obtained from the University of Liverpool before commencing the study.

Previous Forum event attendees and mailing list members (~800) were emailed with a study information sheet and flyer as part of monthly newsletters between November 2023 and April 2024. Interested potential participants had to contact a research team member via email if they were interested in taking part. People with dementia were checked for mental capacity by a research team member before taking consent by discussing the study in a video call and asking the person with dementia to explain in their own words what the study is about, the anonymous and voluntary nature of the study, and the ability to withdraw from participation at any time without having to provide a reason. This was based on the Mental Capacity Act (2005). People with dementia deemed to have mental capacity were then invited to complete the consent form and participate.

### 2.2 Data Collection

Semi‐structured interviews were conducted by three postdoctoral researchers (J.C.W., M.R., M.P.), who were part of the Forum, remotely via Zoom and audio recorded via the Zoom software. The semi‐structured topic guide (Supporting Information) was co‐produced with a public advisor, who is a former carer (H.T.). Questions focused on the level of engagement and involvement in the Forum, the experiences of being involved and networking, learning new information, and any other impacts of the Forum. Audio files were transcribed by a University‐approved transcriber and anonymised.

### 2.3 Data Analysis

Data were analysed by three research team members (C.G., J.C.W., H.T.) using inductive thematic analysis, utilising the six‐step approach by Braun and Clarke [15]. This involved familiarising oneself with the anonymised transcripts and data and generating codes separately before meeting to discuss potential themes jointly. These generated themes were then discussed with the wider team and jointly reviewed and named.

### 2.4 Public Involvement

As part of this Community of Practice evaluation, one unpaid carer (H.T.) was a public advisor as part of the study team. They helped to develop the questions for the interview topic guide and were supported in coding two anonymised interview transcripts. The public advisor also provided feedback on interpreting the findings, and will produce a lay summary for the NIHR Applied Research Collaboration North West Coast with support. They were renumerated for their time.

## 3 Results

### 3.1 Participants

A total of 17 participants were interviewed for this evaluation. The majority (*n* = 13) were female (76.5%) and White British (*n* = 15, 88.2%). Most resided and/or worked in the North West of England (*n* = 13; 76.5%). In terms of their background in dementia, some participants discussed their involvement as multifaceted, both as carers/former carers and as leaders, or members of other organisations focused on people with dementia and unpaid carers. As such, some participants may be noted as both carers and working for charitable support organisations. Of the participants who were current or former unpaid carers for people with dementia (*n* = 7; 41.2%), four had additional roles as either public advisors or the running of charitable or support organisations for people with dementia and unpaid carers. Several participants in this study were involved in the Forum in a singular capacity—including three unpaid carers. There were also two participants (11.8%) who deliver community social support and events for people with dementia and carers, two (11.8%) who are leaders or members of charitable organisations, two (11.8%) healthcare workers (one mental health nursing student and an occupational therapist) and three University academics (17.6%) interviewed. One person with dementia participated in the study.

### 3.2 Qualitative Findings

Four overarching themes were identified in the data: facilitated networking within and outside of personal expertise, improved knowledge and capacity, empowerment by having one's voice heard, and diversity in stakeholders but not the background.

#### 3.2.1 Facilitated Networking Within and Outside of Personal Expertise

Engaging in the Forum facilitated increased networking beyond one's own networks and personal or professional background. By having diverse audiences in the Forum, including health and social care practitioners and people with lived experiences of dementia, unpaid carers, for example, were able to directly connect with academics and care home workers, or Third Sector representatives could connect with commissioners and local authority staff or psychologists. This border crossing of traditionally held separate professions in an informal environment with a clear topical focus—dementia and ageing—thus enabled individuals to expand their networks and learn more about the field as a result.

I think it's probably expanded my network…particularly with sort of colleagues, who maybe have a similar role to me…which has been useful to sort of make those make those links. I suppose what I've got out of it as well…it's hard to beat speaking to or hearing from people with that actual lived experience in whatever capacity it might be, because it really helps sort of ground what you're working on really. It gives it a bit more authenticity and makes it more relevant and you might make what appears to be quite small changes but actually, incredibly important to make it much more relevant ultimately.

(Participant 11, Academic Lecturer)

People with lived experiences felt positive about being brought together with people with high decision‐making power for example at one of the Forum events. By removing the hierarchy of people's backgrounds, the Forum facilitated informal and easy networking which may not be possible in standard working environments.

I actually felt that was worth attending [forum seminar with Member of Parliament delivering talk], very much worth attending, because you've actually got somebody, an MP, who hopefully can help to make a difference because we can all say and do and work, but if the people who have the power aren't on board then you know some of the work [research, networking etc. of the forum], that good, really good work that's being done can be lost.

(Participant 7, Former unpaid carer)

Being involved in the Forum also helped people to develop their organisations and aided in supporting people with dementia and unpaid carers. The Forum offers regular opportunities for non‐academic speakers to get involved, providing platforms to diverse audiences which may be difficult to achieve within set working parameters otherwise.

I was given an opportunity…to do a brief talk on the work of the Dementia Action Alliance which was great. Network meetings which occasionally just asked a few people for any news that we have and I've been included in that so I've been able to pass on news and it's been great sometimes. I've got feedback from some of those attending have followed up with me saying, ‘that's interesting’, they wanted to know more and other times I've just been an audience member.

(Participant 14, Former unpaid carer)

As a result of making new connections at the Forum, knowledge has been shared between attendees outside of Forum events. This showcases how networks were built upon after events, with implications for not only the attendees but also the specific groups and organisations they represent. One attendee, for example, was able to connect with an Admiral Nurse who subsequently attended their group to share information about the Admiral Nurse service and how to access this.

…it's [the Forum] also put me in touch with other professionals and particularly at the conference I met an Admiral Nurse and that has been a fantastic link to make…They're incredibly helpful and knowledgeable and our ability to access that type of support is so limited… that Admiral Nurse is going to come to our session and hold a clinic, so they'll [social group attendees] get first‐hand information which will be fantastic.

(Participant 12, Owner/Manager Social Group)

Engaging with the Forum also helped raise awareness about specific issues in dementia without directly talking at events. By attending events, such as the annual conference, information about specific topics could be shared by expanding one's network. One such example of a specific subtype of dementia is outlined below:

…if I've got availability I will take any opportunity…it's a really good, very cost effective way to raise awareness of Lewy Body [dementia], especially at the last conference [in Liverpool, 2023] you know, to have a stand…more people came to the stand, not just because we have the game [Dementia Inequalities Boardgame]…more people came, had a conversation and I came away with some great connections.

(Participant 2, Charity CEO and former unpaid carer)

#### 3.2.2 Improved Knowledge and Capacity

Joining webinars and other Forum events, such as regional networking meetings, improved knowledge about dementia in attendees from diverse backgrounds. Rare dementia was one topic covered in events that recurred frequently, including a focus on speech‐related dementias. Talks also included foci on other rare dementias, such as Lewy Body and frontotemporal dementias. Participants shared how they could learn about these topics by joining Forum events. This included care professionals caring for people with dementia, allowing them to understand better the nuances of behavioural, linguistic, cognitive and everyday functioning symptoms in dementia.

Yes, I went online for that one because as I soon as I saw Primary Progressive Aphasia as one of the topics [husband diagnosed with PPA]. I mean I don't know her bit as soon as it said UCL I thought well this is what I need to know, I need to you know be there for [the webinar].

(Participant 1, Unpaid carer)

I went to the one [webinar] around from the speech therapist telling us about a different way of assessing [people with dementia] and process and thinking about language skills…So, if I'm going out and doing an assessment [physical assessment for a person with dementia], actually that has a direct impact even now straight away listening to that webinar. Going, ‘oh, ok, maybe I need to explore a little bit more here around their speech’…and so often, I'm looking at function and ability., but that was highlighting their functional ability may decline later on [in dementia]…in turn has led to lots of conversations within our community team…to explore it a bit further and actually have these conversations.)

(Participant 5, Occupational Therapist)

Students also saw the potential of the Forum to increase knowledge in the workforce, identifying a need for the care workforce to receive more training and access to information about dementia. The Forum could be utilised as a way to increase staff education.

…in my own experience, when I used to work in a nursing home and around the dementia patients, and they [nursing home staff] mistook them, they said they're [person living with dementia] aggressive or you know they don't want to take their meds…I think they don't understand some of…the symptoms…that dementia people have you know, so they mistook that for some other kind of mental problem, which you have to differentiate dementia from mental [health issues]…I tried to teach myself to recognise, for instance, when a dementia person gets aggressive, to find out why, because sometimes they can't explain what happen[ed]. So, you have to find out why ‐ if they're too hot, if they're too cold, if they're hungry, if they're thirsty….

(Participant 13, Nursing Student)

Those participants who were newer to the Forum were keen to become more engaged, attend events and increase their knowledge about dementia and ageing. This could be achieved by attending different types of events and showed the growing recognised value of the Forum to provide information.

I am really interested as I say to [get involved], also anything that can help people going through it [dementia], because you've got to realise now, [my] mother's sister has got dementia, two brothers had dementia…you know we all talk about it all the time.

(Participant 16, Unpaid carer)

#### 3.2.3 Empowerment by Having One's Voice Heard

Attending Forum events was described as creating a more equal platform where opinions and experiences about dementia and ageing can be shared, removing the standard hierarchy of patient and clinician, for example. By creating an informal and supportive environment, the Forum events have helped to empower people who may not normally feel heard, such as unpaid carers and people living with dementia. Whilst carers may have previously been nervous to engage in some instances, the Forum supported them to share their opinions and experiences.

…the first time I spoke [at a forum event] I was like, ‘this is going to be awful and nobody's going to be interested’, because they will all know better because they know the clinical side of things. But, the first one [talk] I did was with another former carer and we had 2 completely different caring journeys, and it sort of opened people's eyes as to how you can be treated in one area as to another area [geographic inequalities].

(Participant 8, Former unpaid carer)

By offering anyone with a background in dementia and ageing to engage and share their experiences, unpaid carers were able to share their stories and their care experiences along care pathways. This provided important learning for health and social care professional staff, and for other carers. One participant, an occupational therapist, noted their ability to be the voice of people with dementia who may not be involved in research or the Forum. Moreover, the Forum was described as developing knowledge through talking to other practitioners and people with lived experiences, which can impact service delivery improvements.

my interest in…research side of things and keeping abroad of what's going on…that affects my patient group and therefore then in turn could affect my practice…doing the best for my client group. So, the only way is to be informed is to find out what research is happening and being involved in that research as much as possible…I am hearing people's experiences [people with dementia and unpaid carers] and whilst there are people who do get involved in research who have the condition…an awful lot of people don't and therefore as a practitioner I can be that voice…I can hopefully give some something back to the research that can develop the future practice and things.

(Participant 5, Occupational Therapist)

This was also the case for unpaid carers. Carers highlighted the benefits of being able to discuss not only general issues and challenges in dementia and service access, but also in using knowledge to help others and support people who may be in a similar situation.

I've been involved with the carers voice and the carers action group for about 8 years now. So, over them years we've met with MPs, care commissioners, social services and we've brought our concerns. We've told them about the challenges we faced. I've discussed when I couldn't get the help with them [services] so when [a friend] got in touch with me and said, ‘Clarissa [Liverpool University and LDARF] was doing research into dementia’ specifically, that just that was like a lightbulb moment for me. I had so much I wanted to share, and I want to make it better for my dad [living with dementia] and for everyone else and for carers as well. I don't want anyone to go through what me and my family went through.

(Participant 4, Unpaid carer)

Empowerment within Forum events has also led some attendees to champion the Forum and its work outside in other organisations, settings and groups. Many unpaid carers, some of whom were also public advisors on research and thus contributed to developing, shaping, conducting and disseminating research, felt more knowledgeable and vested in passing on knowledge gained in the Forum to outside people and organisations, including fellow carers.

…it's [being involved in the forum] enabled me to have the knowledge to say to people, ‘oh you want to try this, and this might be good for you’, you can talk with a little bit more knowledge. I've had the research element and the knowledge from the meetings in scenarios like that… you never know the moment when you can pass something on [information or advice] and sometimes it goes the other way…from [the] caring role and passing forward to our dementia researchers, as opposed to coming from somebody speaking at a conference…So you learn things from different areas and pass it forward to others.

(Participant 15, Former unpaid carer, public advisor)

Participants reported how, for someone with lived experiences of dementia, hearing about research and different opportunities of becoming involved, either as a participant or a team member and public advisor for the first time, usually when attending their first Forum meeting, they felt encouraged to get involved. By becoming involved in research, one person with young‐onset dementia felt that this would be a further opportunity to raise awareness of dementia not being a condition that only affects people aged 65 or older.

I'm really glad I found the forum and I can be part of things like research, and just go and give my point of view from being somebody that's not got a recognised form of dementia and being somebody younger. I think that's my way of bringing awareness to, you know, you don't have to be elderly [to be diagnosed with dementia]*.*

(Participant 3, Person living with dementia)

#### 3.2.4 Diversity in Stakeholders But Not Background

Being a part of the Forum has helped to foster relationships with people from both similar and different backgrounds. The Forum opened up varied experiences to attendees, courtesy of both the reach of the Forum geographically and across different stakeholders and the breadth of the groups and societal backgrounds of the people involved. Many of the participants noted in particular that the Forum was diverse in its membership and in terms of the voices it helped to amplify throughout its events. For example, one participant and Forum member felt that the diversity of the membership and inclusion of people from varied backgrounds was one of its key strengths.

I do think that's one of its strengths [representativeness of forum membership], because it does include a wide range of people from different organisations and settings and professions, lay people as well if I can use that term, it's not just people working in the field, so I think it's a very open and inclusive forum.

(Participant 9, Vice‐Chair of Dementia Advocation Group)

Another participant and Forum member felt that being able to network and discuss dementia‐related issues and experiences with people from different backgrounds, including clinical, academic and lived experience, was a great opportunity for members to learn from each other and pass on valuable information.

So yes, there was good opportunity to mix and talk with people from different interests or different expertise… thinking about the conference and the way that worked…I think it was a good mix…Also I was chatting to people from sort of services, health service and managers and clinicians. So, you know it was good to get that mix of clinical practice or social care practice and research.

(Participant 17, Academic)

However, some participants also noted some current limitations of the Forum membership. In particular, one participant discussed the fact that the same voices are often heard at events, with limited availability for input from new members or different members with new or potentially novel experiences and insights. They also discussed the need for more varied socio‐demographic backgrounds among Forum members, particularly among those with lived experience, including people with dementia and unpaid carers. This can reflect the relative sense of empowerment among those in positions of relative authority or larger groups, or confidence to contribute. There was a suggestion that the Forum appeared to be mostly engaged by people from White, middle‐class backgrounds, which is not reflective of the wider experiences of other societal groups.

…we've got people with lived and professional experience and the same cannot be said for a lot of groups like this [the forum]. But when we look at the people that take part in our events, they are what I would refer to as a bit of an elite sample of people because these are people who are quite proactive. It's a certain breed of person, there's a certain type of person that takes part in stuff like this and they are typically white middle class. I don't think we're that representative of other ethnicities. But that's a sector‐wide issue and it's something that we're aware of and it's unfortunate, because I think the whole theme [of the forum] is around inequalities and whilst I don't think for one second we are promoting inequality, I just think there's always room for improvement in terms of representation in these things.

(Participant 10, Academic)

If it's something new and innovative I'm all ears, I am there for listening. Really enjoyed the information about the things going on in…the Netherlands…they have an innovative approach to things [care for people with dementia] and I know things are going over here now that we're trying to copy. I've enjoyed taking part in discussions where people put forward their views and points of standing on the dementia issue. What does get me is that the same people stand up and say the same things at the same meetings over and over again… I do feel as though people's time needs to be used properly and it doesn't mean listening to people saying the same things all the time and I have to say it usually comes from people who are carers.

(Participant 6, Unpaid carer, NHS Governor)

## 4 Discussion

The Liverpool Dementia & Ageing Research Forum provides a platform to connect, develop knowledge and skills, and share learnings about dementia and ageing across diverse stakeholders. The Forum is the first evidenced Community of Practice to connect experts from different backgrounds in the field of dementia and ageing, as opposed to staying within one type of profession such as Dementia Champions [13].

Raising capacity and improving knowledge of dementia and ageing in the care workforce is of particular need, given continued evidence of the lack of workforce knowledge [16]. Both people with dementia and their carers [17] as well as the health and social care workforce [18] have reported the shortcomings of workforce education on the topic area, including a lack of link‐up between health and social care, knowledge about dementia subtypes and end of life care, and lack of cultural understanding to support people with dementia and their families well. Whilst targeted training is vital to educate the workforce from the start and throughout their careers, creating a peer and stakeholder community with a shared interest in the topic area to discuss experiences and new services and knowledge directly from research, such as via the Liverpool Dementia & Ageing Research Forum, can create an additional layer of informal ‘soft’ education that can help to fill some of the gaps in workforce knowledge. A recently published study by Latham et al. [19] corroborates the value of peer support learning and non‐formal training provision (such as via the Forum). Latham et al. [19] reported how informal learning took place by care workers working with people with dementia via their peers, available resources and cultural knowledge within the care home, showing how formal training can only offer some level of influence on care practices. Thus, a cross‐organisational and ‐stakeholder network such as the Forum can provide more diverse and representative informal learning to educate the care workforce and others.

Improving knowledge and building capacity is not only relevant to the care workforce [16]. Many people with dementia and their unpaid carers feel inadequately supported and informed about the dementia diagnosis, as well as lacking adequate knowledge before a diagnosis to approach the General Practitioner (GP) about symptoms in the first place, which is supported by GPs’ points of view [2, 4]. Thus, by providing information both about symptoms to help people approach healthcare and social care professionals and about the health and social care system and how to navigate this, many people with lived experiences who participated in this study were found to be empowered.

Empowering people with dementia and unpaid carers was possible by creating an inclusive and supportive environment. Participants felt included and able to share their experiences and views in the Forum, including towards healthcare professionals and academics—groups of professionals who may usually not be available to engage with. Connecting people from different professional and personal backgrounds with a shared focus on dementia and ageing facilitated cross‐boundary discussions, learning and networking. Aligned with McConnell's co‐produced definition of empowerment in dementia, people with dementia felt their confidence boosted, whilst being respected and having their voices heard to create change and make an impact [20]. Whilst the stakeholder backgrounds were noted as diverse, such as psychologists, people with dementia, third‐sector providers and care home managers, other areas of personal diversity, such as ethnic background and young‐onset dementia, were not well represented in the Forum to date. Drawing on limited evidence on hard‐to‐reach groups in dementia and ageing research, many research studies also face a lack of true diversity due to various factors. Field et al. [22], for example, report difficulties in adjusting to a dementia diagnosis for people to come forward and take part, whilst Liljas et al. [21] reported that ethnic background and existing support within their social networks, as well as poverty, led to reluctance in engaging in health promotion activities in older adults. To ensure wider representation and shared experiences in the Forum though, more work needs to be done to go out into communities and inform people of the Forum and facilitate easy access. However, concerns expressed by some respondents about dominant voices within meetings highlight the delicate balance in a wide stakeholder Community of Practice of enabling people to be encouraged to contribute by accommodative meeting leadership and chairing, whilst also discouraging dominating voices to facilitate those contributing less actively to do so. Equity of esteem is essential but needs supplementing with techniques to actively encourage wider contributions.

Whilst the study benefitted from diverse stakeholders in the field of dementia and ageing who participated, including people with lived experiences of dementia, healthcare and social care professionals, Third Sector representatives and academics, there are some limitations to note. First, due to the inclusive nature of the Forum, postdoctoral research interviewers were also members of the Forum, which may have biased the experiences shared. However, participants shared suggestions for modifying and further improving the Forum also, without feeling they were unable to share this. Despite advertising for participation in the study, only one person living with dementia participated in the study. It was not possible to approach people who are members of the Forum directly, but instead generic recruitment advertisement was circulated several times via the Forum, requiring interested potential participants to approach the study team. However, the lack of direct voice of people with dementia in the study is reflected in the membership of the Forum, with fewer people with dementia represented compared to the number of healthcare or social care professionals or unpaid carers. Moreover, this study lacked ethnic diversity representation, which is also less represented in the Forum itself. Given that only Forum members were invited to take part, it was not surprising to find a lack of ethnic diversity therefore in the study participants. Thus, as a next step, the Forum needs to conduct more outreach work to ensure greater diversity of its membership by having greater representation of people living with dementia and people from minority ethnic backgrounds involved.

## 5 Conclusions

The Forum offers an innovative and engaging format to learn, build capacity and network in the field of dementia and ageing, bridging the divide between care professionals, academia and lived experts in this Community of Practice. The Forum can be used as a knowledge and capacity‐building model for dementia in different regions, but also in different countries and population groups, such as learning disabilities, obesity or motor neuron disease. Ongoing developments are in progress to implement the Forum in the state of Victoria, Australia, for example, which will need particular considerations regarding the region's rurality. In view of often limited workforce training on the job, this Community of Practice provides a suitable and effective form of knowledge exchange for the workforce and other stakeholders in the field.

## Author Contributions

**Clarissa Giebel:** conceptualisation, investigation, writing–original draft, methodology, formal analysis. **James Watson:** conceptualisation, writing–review and editing, data curation, formal analysis. **Megan Polden:** data curation, conceptualisation, writing–review and editing. **Megan Readman:** data curation, conceptualisation, writing–review and editing. **Hilary Tetlow:** conceptualisation, writing–review and editing, formal analysis. **Mark Gabbay:** conceptualisation, writing–review and editing, formal analysis.

## Disclosure

The views expressed in this publication are those of the authors but not necessarily those of the National Institute for Health and Care Research or the Department for Health and Social Care.

## Ethics Statement

Ethical approval was obtained from the University of Liverpool ethics committee (Ref: 12906) before commencing the study.

## Conflicts of Interest

The authors declare no conflicts of interest.

## Supporting information

Supporting information.

## Data Availability

The data that support the findings of this study are available on request from the corresponding author. The data are not publicly available due to privacy or ethical restrictions.
