# Supplementary material for: Engaging With a Community of Practice in Dementia: Impacts on Skills, Knowledge, Networks and Accessing Support
Source: Health Expect. 2025 Jan 15;28(1):e70154. doi: 10.1111/hex.70154 (PMC11735733; doi:10.1111/hex.70154)
Supplement: Supplementary file 1 — Supporting information. [file HEX-28-e70154-s001.docx]

**APPENDIX I - TOPIC GUIDE**

1. To start us off, tell us a bit about yourself – what is your experience with dementia?

2. How have you been involving with the Liverpool Dementia & Ageing Research Forum and since when?

3. What type of events have you attended and why did you choose those?

4. What have you learned from engaging with the Forum in general, and has it affected your network and who you know in the field of dementia?

5. If you live with dementia/ care for someone/ provide care for people with dementia, has engaging with the Forum impacted on your care/care delivery?

6. How have you been involved with research from the Forum?

7. Do you feel comfortable sharing your opinions and thoughts at the Forum events and research? Do you feel your voice and thoughts have been adequately taken on board and made a difference?

8. Thinking about different experiences and backgrounds, do you feel the Forum and research are reflecting diversity?

9. What have you learned from engaging with research discussions and/or activities from the Forum, have you learned new skills? Have you been able to apply them or how would you like to do so?

10. Is there anything else about your experiences with the forum that you would like to share?
